# Intensity of Rainfall and Severity of Melioidosis, Australia

**DOI:** 10.3201/eid0912.020750

**Published:** 2003-12

**Authors:** Bart J. Currie, Susan P. Jacups

**Affiliations:** * Menzies School of Health Research and Flinders University, Darwin Northern Territory, Australia

**Keywords:** Melioidosis, *Burkholderia pseudomallei*, Epidemiology, Bacterial ecology, Pneumonia, Septic shock

## Abstract

In a 12-year prospective study of 318 culture-confirmed cases of melioidosis from the Top End of the Northern Territory of Australia, rainfall data for individual patient locations were correlated with patient risk factors, clinical parameters, and outcomes. Median rainfall in the 14 days before admission was highest for those dying with melioidosis (211 mm), in comparison to 110 mm for those surviving (p = 0.0002). Median 14-day rainfall was also significantly higher for those admitted with pneumonia. On univariate analysis, a prior 14-day rainfall of ≥125 mm was significantly correlated with pneumonia (odds ratio [OR] 1.70 [confidence interval [CI] 1.09 to 2.65]), bacteremia (OR 1.93 [CI 1.24 to 3.02]), septic shock (OR 1.94 [CI 1.14 to 3.29]), and death (OR 2.50 [CI 1.36 to 4.57]). On multivariate analysis, rainfall in the 14 days before admission was an independent risk factor for pneumonia (p = 0.023), bacteremic pneumonia (p = 0.001), septic shock (p = 0.005), and death (p < 0.0001). Heavy monsoonal rains and winds may cause a shift towards inhalation of *Burkholderia pseudomallei.*

Melioidosis, infection with *Burkholderia pseudomallei,* is endemic in Southeast Asia and northern Australia (*1*). Within the disease-endemic region, reported incidence has been increasing; melioidosis is now recognized as the most common cause of severe community-acquired sepsis in parts of northeast Thailand (*2*) and the most common cause of fatal community-acquired bacteremic pneumonia in the tropical “Top End” of the Northern Territory of Australia (*3*). The recognized endemic region for melioidosis has also been expanding, with recent reports from Taiwan (*4*), China, and India (*1*). Sporadic foci of melioidosis have occurred in temperate locations, possibly resulting from introduced infection (*1*,*5*). Melioidosis is also an important infection to consider in travelers returning from a disease- endemic region (*6*,*7*). While most cases are from recent infection with *B. pseudomallei*, latency is well recognized, and disease has occurred up to 29 years after a person has left a melioidosis-endemic area (*8*).

The association between rainfall and melioidosis has long been recognized, with 75% and 85% of cases occurring in the wet season in northeast Thailand (*9*) and northern Australia (*3*), respectively. In both regions, the number of seasonal cases correlates with total rainfall.

*B*. *pseudomallei* is an environmental bacterium of soil and surface water in disease-endemic locations. We have previously documented the incubation period for melioidosis from defined inoculating events to be 1–21 (mean 9) days (*10*). While most cases are considered to be from percutaneous inoculation (*10*,*11*), inhalation is also well recognized as a mode of infection. We have noted that melioidosis patients are often more severely ill after heavy monsoonal rainfall. We now show that intensity of rainfall is an independent predictor of melioidosis in persons admitted to hospital with pneumonia and of death. We postulate that heavy rainfall results in a shift towards inhalation as the mode of infection with *B. pseudomallei,* which leads to more severe illness.

## Methods

### Patients

The Darwin prospective melioidosis study has documented 318 culture-confirmed cases of melioidosis that occurred in the Top End of the Northern Territory in the 12 years from October 1989 until October 2001. Patient data are stored in Oracle software, version 8.0.4 (Oracle, North Sydney, Australia). Patient variables, as defined previously (*3*), include age, sex, ethnicity (aboriginal, non-aboriginal), location, and risk factors, including diabetes, alcohol excess, chronic lung disease, smoking, chronic renal disease, and kava use. Clinical parameters include nature of primary melioidosis signs and symptoms (pneumonia, other), presence of bacteremia, septic shock (presence of hypotension not responsive to fluid replacement together with hypoperfusion abnormalities manifest as end organ dysfunction) (*12*), and outcome (death, survival).

### Rainfall Data

The Top End covers 516,945 km^2^. Daily rainfall data from 12 recording stations, located throughout the region and including major remote Aboriginal communities, were provided by the Bureau of Meteorology in Darwin. From these data we calculated the rainfall at each patient’s location for defined periods before date of admission. Given a mean incubation period of 9 days for acute melioidosis, we used rainfall in the 14 days before admission for each patient (14-day rainfall) to broadly reflect the rainfall exposure around the infecting event.

### Statistical Analysis

Statistical analyses were performed by using Intercooled STATA 7.0 (Stata, College Station, TX). Initially, median 14-day rainfall was compared for patient variables and clinical parameters. Analysis by t tests was performed after the rainfall data were normalized by using square root transformation. Subsequently, univariate and multivariate analysis was performed with the outcomes being the various clinical parameters. Categorical variables included were age (<45 years, ≥45 years), gender, ethnicity, diabetes, alcohol excess, chronic lung disease, smoking, chronic renal disease, kava use, absence of any risk factors (those listed above or age ≥45 years or cardiac failure, malignancy, or immunosuppressive therapy) and 14-day rainfall (<125 mm, ≥125 mm). Separate multivariate analysis was also performed with normalized 14-day rainfall data as a continuous variable. All logistic regressions were performed by using stepwise forwards technique to find the most parsimonious and significant model.

## Results

The Figure shows the close association between total monthly rainfall, as recorded at Darwin Airport, and the number of cases of melioidosis in the Top End for each month during the 12 years. The correlation between monthly cases of melioidosis and rainfall at Darwin Airport in the preceding calendar month (r = 0.617; p < 0.0001) was slightly tighter than the correlation with rainfall in the concurrent month (r = 0.574).

**Figure:**
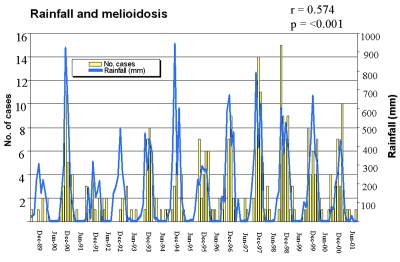
Monthly rainfall and melioidosis cases during 12- year study period, Australia.

Table 1 shows the median 14-day rainfall correlation with various risk factors, clinical signs and symptoms, and outcomes. The correlation with median 14-day rainfall was significantly higher for patients with pneumonia, those with bacteremia and septic shock, and those who died. For those patients with pneumonia, correlation with median 14-day rainfall was significantly higher if they were bacteremic. Patients with diabetes, alcohol excess, and chronic renal disease were all associated with significantly higher median 14-day rainfall; correlation with 14-day rainfall did not significantly differ for age, sex, ethnicity, chronic lung disease, and smoking (data not shown).

**Table 1:** Prior 14-day rainfall correlations with risk factors, clinical signs and symptoms, and outcomes

Parameter	Yes		No		p value
	No.	Median 14-d rainfall	No.	Median 14-d rainfall	
Diabetes	119	174 mm	199	102 mm	0.002
Alcohol excess	118	132 mm	200	116 mm	0.043
Chronic renal disease	27	198 mm	291	113 mm	0.048
Pneumonia	156	161 mm	162	105 mm	0.001
Bacteremia	155	166 mm	163	97 mm	<0.0001
Pneumonia–bacteremic	86	188 mm	70	120 mm	0.035
Nonpneumonia–bacteremic	71	136 mm	91	89 mm	0.007
Septic shock	74	191 mm	244	112 mm	0.0008
Death	56	211 mm	262	110 mm	0.0002

Table 2 shows that, on univariate analysis, 14-day rainfall ≥125 mm correlated significantly with primary symptoms of pneumonia, bacteremia, and septic shock, and with death. Table 3 shows independent predictors of clinical signs and symptoms and outcome when multivariate analysis with 14-day rainfall as a categorical variable was used. When 14-day rainfall was used as a continuous variable, it was an independent risk factor for a admission with pneumonia (p = 0.023), bacteremic pneumonia (p = 0.001), septic shock (p = 0.005), and of death (p < 0.0001). Notably, while absence of any risk factors was a predictor of primary signs and symptoms other than pneumonia and of less severe disease, neither diabetes, alcohol excess, nor chronic renal disease was an independent predictor of signs and symptoms, disease severity, or death.

**Table 2:** Univariate analysis for correlation with prior 14-day rainfall of ≥125 mm

Clinical parameter		14-d rainfall ≥125 mm	14-d rainfall <125 mm	Odds ratio (95% CI)^a^	p value
Pneumonia	Yes	88	68	1.70 (1.09 to 2.65)	0.019
	No	70	92		
Bacteremia	Yes	90	65	1.93 (1.24 to 3.02)	0.004
	No	68	95		
Bacteremic pneumonia	Yes	53	33	1.94 (1.17 to 3.21)	0.010
	No	105	127		
Septic shock	Yes	46	28	1.94 (1.14 to 3.29)	0.014
	No	112	132		
Death	Yes	38	18	2.50 (1.36 to 4.57)	0.003
	No	120	142		

^a^CI, confidence interval.

**Table 3:** Multivariate analysis of predictors of clinical signs, symptoms, and outcome

Clinical parameter	Independent predictors	Odds ratio (95% CI)^a^	p value
Pneumonia	Smoking	2.51 (1.51 to 4.18)	<0.0001
	Prior 14-d rainfall ≥125 mm	1.58 (0.96 to 2.59)	0.069
	Kava use	0.28 (0.09 to 0.82)	0.020
	Absence of risk factors	0.29 (0.13 to 0.66)	0.003
Bacteremic pneumonia	Aboriginal ethnicity	2.28 (1.33 to 3.90)	0.003
	Prior 14-d rainfall ≥12 5mm	1.81 (1.06 to 3.10)	0.031
	Absence of risk factors	0.06 (0.01 to 0.48)	0.007
Septic shock	Prior 14-d rainfall ≥125 mm	1.71 (0.99 to 2.97)	0.057
	Absence of risk factors	0.07 (0.01 to 0.55)	0.011
Death	Prior 14-d rainfall ≥125 mm	2.48 (1.32 to 4.66)	0.005
	Smoking	1.93 (1.00 to 3.72)	0.050
	Absence of risk factors	No deaths in this group	<0.0001

^a^CI, confidence interval.

## Discussion

Our data confirm our observations that patients admitted with melioidosis 1–2 weeks after heavy monsoonal rainfall are more ill and more likely to die. Median rainfall in the 14 days before admission was highest in those who died with melioidosis (211 mm). For those admitted with bacteremic pneumonia, prior 14-day median rainfall was 188 mm, compared with 89 mm in patients who were nonbacteremic and did not have pneumonia. Multivariate analysis showed that rainfall in the 14 days before admission was an independent predictor of septic shock and death. Patients were 2.5 times more likely to die from melioidosis if the rainfall in the 14 days before admission was ≥125 mm. Overall, 68% of deaths occurred in this high rainfall group. Furthermore, prior heavy rainfall was an independent predictor of admission with pneumonia rather than with no pneumonia. Patients were almost twice as likely to have bacteremic pneumonia if the rainfall in the 14 days before admission was ≥125 mm.

Earlier literature, including that involving soldiers from the Vietnam War, suggests that inhalation is a common mode of infection with *B. pseudomallei* (*13*,*14*). This scenario was proposed for those exposed to dust raised by helicopter rotor blades in Vietnam (*15*). However, recent reviews have supported the predominant role of percutaneous inoculation of *B. pseudomallei* after exposure to muddy soils or surface water in endemic locations (*10*,*11*,*16*). Admissions with melioidosis pneumonia after presumptive inoculating skin injuries have been documented in patients with soil-contaminated burns (*17*) and are also common in our hospital (*3*,*10*). This finding suggests hematogenous spread to the lung rather than inhalation or spread from the upper respiratory tract. This finding is analogous to postprimary tuberculosis, with disease from hematogenous spread localizing in the upper lung zones, where highest alveolar oxygen tension exists (*18*). Moreover, septicemic melioidosis pneumonia patients are often more systemically ill than is suggested by initial chest x-ray, supporting the concept of spread to, rather than from, the lung.

Even if percutaneous inoculation is more common overall, the association of prior heavy rainfall with both pneumonia and more severe disease may well reflect a shift towards inhalation as the mode of acquiring *B. pseudomallei*. The periods of intense monsoonal rainfall are usually also associated with heavy winds and melioidosis cases, and outbreaks are documented after cyclonic winds and rain (*19*,*20*). Aerosolization of bacteria from surface soil and water under such conditions is probable, resulting in the potential for inhalation of *B. pseudomallei*. Melioidosis following near-drowning is well recognized, with aspiration considered the likely infecting event, followed by pneumonia after an incubation period as short as 2 days (*21*–*23*).

That melioidosis can potentially be more severe after inhalation than after percutaneous inoculation is not surprising. This finding is well recognized for anthrax, plague, and tularemia and has implications for biological warfare considerations (*24*–*26*). However, as with melioidosis, septicemia with pulmonary involvement after percutaneous inoculation is well recognized with anthrax, plague, and tularemia. The lack of clarity of correlation between mode of infection, site of disease, and clinical course in the melioidosis literature is also evident in descriptions of the closely related disease, glanders (infection with *B. mallei*) (*15*).

Additional possible explanations for more severe disease after heavy rainfall include a larger bacterial inoculating dose and infection with more virulent bacteria. The association of melioidosis with the wet season has also been postulated to be due to movement of *B. pseudomallei* from deeper soil layers to the surface with the rising water table (*27*). Early studies also speculated that the increased isolation of *B. pseudomallei* from surface water after heavy rains resulted from increased growth of the bacteria (*28*). More recently, the possibility has been raised that *B. pseudomallei* may persist in the environment in a viable nonculturable state during times of stress, such as in prolonged dry seasons (*20*,*29*). Differential gene activation likely allows such environmental bacteria to respond and adapt to different environmental conditions (*30*). Recently, viable but nonculturable cells of *Francisella tularensis* have been shown to be avirulent in mice (*31*). Thus, both increased environmental bacterial load and increased virulence of environmental *B. pseudomallei* may possibly result from periods of heavy rainfall. A possible confounder to analyzing associations of rainfall with disease severity is that, whatever the mechanisms of more severe disease, such cases will tend to have shorter incubation periods. Therefore, the prior 14-day rainfall is likely to more closely reflect the rainfall associated with infection in these cases than in less severe cases, where incubation periods >14 days might occur.

In patients with melioidosis in this study, diabetes, alcohol excess, and chronic renal disease were associated with higher prior rainfall. We previously suggested that the predisposition to melioidosis in persons with these three conditions may relate primarily to impaired polymorphonuclear leukocyte (PMNL) functions (*3*). This hypothesis is supported by data from an observational, uncontrolled study showing improved survival with use of granulocyte colony-stimulating factor (G-CSF) in melioidosis septic shock (*32*). Recent animal data suggest an important role for lung-derived G-CSF in controlling intrapulmonary infection (*33*). Therefore, in diabetes, alcoholism, or chronic renal disease, both impaired phagocytic activity of alveolar macrophages and impaired recruitment of PMNL into the lungs as a result of acquired dysfunction of alveolar macrophages may be critical, in addition to impaired PMNL function, in determining the predisposition to melioidosis pneumonia. Such a predisposition is likely to be especially important in influencing whether infection becomes established after inhalation of *B. pseudomallei*. The association of diabetes, alcohol excess, and chronic renal disease with higher prior rainfall may therefore reflect a particular susceptibility to inhalation as a mode of infection in patients with these risk factors. Alternatively, this finding may reflect a greater influence of bacterial load or organism virulence in these risk groups. Either explanation is consistent with the observation from Thailand that risk factors and level of environmental exposure to *B. pseudomallei* have a compound interaction, as is evident in the especially high rates of melioidosis in diabetic rice farmers (*34*).

We have shown that the intensity of rainfall in the 14 days before a person is admitted to hospital with melioidosis is an independent predictor of the patient’s having pneumonia, septic shock developing, and death. We postulate that this may reflect a shift towards inhalation of *B. pseudomallei* as the mode of transmission after heavy monsoonal rains and winds.
